# miR-548d-3p Alters Parasite Growth and Inflammation in *Leishmania (Viannia) braziliensis* Infection

**DOI:** 10.3389/fcimb.2021.687647

**Published:** 2021-06-10

**Authors:** Marina de Assis Souza, Eduardo Milton Ramos-Sanchez, Sandra Márcia Muxel, Dimitris Lagos, Luiza Campos Reis, Valéria Rêgo Alves Pereira, Maria Edileuza Felinto Brito, Ricardo Andrade Zampieri, Paul Martin Kaye, Lucile Maria Floeter-Winter, Hiro Goto

**Affiliations:** ^1^ Instituto de Medicina Tropical, Faculdade de Medicina, Universidade de São Paulo (IMTSP/USP), São Paulo, Brazil; ^2^ Departamento de Salud Publica, Facultad de Ciencias de La Salud, Universidad Nacional Toribio Rodriguez de Mendoza de Amazonas, Chachapoyas, Peru; ^3^ Instituto de Biociências, Universidade de São Paulo, São Paulo, Brazil; ^4^ York Biomedical Research Institute, Hull York Medical School, University of York, York, United Kingdom; ^5^ Instituto Aggeu Magalhães, Fundação Oswaldo Cruz (IAM/FIOCRUZ), Recife, Brazil; ^6^ Departamento de Medicina Preventiva, Faculdade de Medicina, Universidade de São Paulo, São Paulo, Brazil

**Keywords:** *Leishmania braziliensis*, microRNA, pathogenesis, active cutaneous leishmaniasis, self-healed cutaneous leishmaniasis, THP-1 cells

## Abstract

American Tegumentary Leishmaniasis (ATL) is an endemic disease in Latin America, mainly caused in Brazil by *Leishmania (Viannia) braziliensis*. Clinical manifestations vary from mild, localized cutaneous leishmaniasis (CL) to aggressive mucosal disease. The host immune response strongly determines the outcome of infection and pattern of disease. However, the pathogenesis of ATL is not well understood, and host microRNAs (miRNAs) may have a role in this context. In the present study, miRNAs were quantified using qPCR arrays in human monocytic THP-1 cells infected *in vitro* with *L. (V.) braziliensis* promastigotes and in plasma from patients with ATL, focusing on inflammatory response-specific miRNAs. Patients with active or self-healed cutaneous leishmaniasis patients, with confirmed parasitological or immunological diagnosis, were compared with healthy controls. Computational target prediction of significantly-altered miRNAs from *in vitro L. (V.) braziliensis*-infected THP-1 cells revealed predicted targets involved in diverse pathways, including chemokine signaling, inflammatory, cellular proliferation, and tissue repair processes. In plasma, we observed distinct miRNA expression in patients with self-healed and active lesions compared with healthy controls. Some miRNAs dysregulated during THP-1 *in vitro* infection were also found in plasma from self-healed patients, including miR-548d-3p, which was upregulated in infected THP-1 cells and in plasma from self-healed patients. As miR-548d-3p was predicted to target the chemokine pathway and inflammation is a central to the pathogenesis of ATL, we evaluated the effect of transient transfection of a miR-548d-3p inhibitor on *L. (V.) braziliensis* infected-THP-1 cells. Inhibition of miR-548d-3p reduced parasite growth early after infection and increased production of MCP1/CCL2, RANTES/CCL5, and IP10/CXCL10. In plasma of self-healed patients, MCP1/CCL2, RANTES/CCL5, and IL-8/CXCL8 concentrations were significantly decreased and MIG/CXCL9 and IP-10/CXCL10 increased compared to patients with active disease. These data suggest that by modulating miRNAs, *L. (V.) braziliensis* may interfere with chemokine production and hence the inflammatory processes underpinning lesion resolution. Our data suggest miR-548d-3p could be further evaluated as a prognostic marker for ATL and/or as a host-directed therapeutic target.

## Introduction

The leishmaniases are vector-borne diseases caused by protozoan parasites of the genus *Leishmania.* Transmitted by *Phlebotomine* sandflies, the leishmaniases are endemic in tropical and subtropical areas, with one million cases/year in 98 countries (Burza et al., 2018). During its life cycle, *Leishmania* exists as promastigotes (elongated forms with an external flagellum) in the sandfly gut and as amastigotes (round or ovoid forms without an external flagellum) within mononuclear phagocytes of the vertebrate host. After promastigote inoculation in the skin by the vector, the parasites interact primarily with tissue humoral and cellular elements and the infection may progress to overt disease. Depending on the *Leishmania* species and host characteristics, the disease may manifest as visceral leishmaniasis, affecting organs, such as the liver and spleen, or tegumentary form, causing lesions in the skin and mucosa. More than 15 species may cause cutaneous leishmaniasis, with *Leishmania (Viannia) braziliensis* the most prevalent species in Brazil, where disease presents as either localized cutaneous leishmaniasis (CL), disseminated cutaneous leishmaniasis, or disfiguring mucosal leishmaniases (Turetz et al., 2002; Machado et al., 2011; Goto and Lauletta Lindoso, 2012). Once diagnosed, most patients are treated with anti-*Leishmania* drugs but rarely the patients heal without any specific treatment. Comparing active cutaneous and self-healed leishmaniasis patients constitutes a unique opportunity to explore pathogenic mechanisms of lesion development and control that are not fully elucidated.

In human CL, lesion development is not directly related to parasite growth, and few parasites are seen in the skin (Sotto et al., 1989). Instead, Th-1-type immune responses essential for infection control also drive inflammation and lesion development and cause tissue damage if uncontrolled (Vieira et al., 2002). In CL lesions characterized by chronic inflammation, activated CD69^+^ T cells (Diaz et al., 2002) and regulatory CD4^+^CD25^+^FOXP3^+^ IL-10–producing T cells, granzyme A CD8^+^ cytotoxic T cells, or even pro-inflammatory CD4^+^ IFN-γ–producing T cells (Bourreau et al., 2009; Faria et al., 2009) have all been observed. In a recent transcriptomic study of skin samples of cutaneous leishmaniasis patients, delayed or absence of cure was correlated with higher expression of gene sets related to the cytolytic pathway, including mRNAs for granzyme (*GZMB*), perforin (*PRF1*), and granulysin (*GNLY*) (Amorim et al., 2019).

microRNAs (miRNAs), endogenous small non-coding RNAs of ~22 nucleotides, have a fundamental role in shaping the host transcriptome (Baltimore et al., 2008) and act as key regulators in gene expression networks, including those regulating cell cycle, mitosis, apoptosis, differentiation, and immune functions. MicroRNAs mediate gene silencing post-transcriptionally by base-pairing to the 3′-untranslated regions (3’UTR) of their respective target genes. Up/down-regulation of miRNA expression impacts various cellular processes during homeostasis but may also result in dysfunction of cellular activities (Bartel, 2004; Bartel, 2009) and participate in pathological processes including infection and inflammation (O’Connell et al., 2012). In the human immune system, miRNA-clusters have been shown to exert essential roles in the regulation of related gene expression, impacting innate and adaptive immune responses (Hirschberger et al., 2018). Furthermore, as most miRNAs are considered stable in biological fluids and resistant to environmental conditions (Sohel, 2016), miRNAs are suitable for evaluation in plasma samples and represent attractive candidates as biomarkers of disease or therapeutic response.

MicroRNAs can be modulated by different pathogens, such as viruses, bacteria, and protozoan parasites (Chandan et al., 2019; Acuna et al., 2020). Differential expression of diverse miRNA has been identified in *Leishmania*-host interaction *in vitro* and experimental *in vivo* systems with visceral and cutaneous strains of *Leishmania* (Acuna et al., 2020) as well as human leishmaniasis (Paul et al., 2020). Specifically, in cutaneous leishmaniasis caused by *L. braziliensis* miR-361-3p, a regulator of GZMB and tumor necrosis factor (TNF) was down-regulated and related to treatment failure (Lago et al., 2018). In contrast, expression of miR-193b and miR-67, involved in regulating expression of triggering receptor expressed on myeloid cells-1 (TREM-1) was positively related to good treatment outcome (Nunes et al., 2018).

In the present study, we searched for differentially expressed microRNA in plasma of patients with active *L. (V.) braziliensis* infection and self-healed CL patients. In addition, we studied *in vitro L. (V.) braziliensis* infected-human monocyte-derived THP-1 cells to provide more direct insights into miRNA function. We focused on miRNA related to immune-inflammatory processes given the role of such processes in CL lesion development and resolution. miRNA expression was found to be markedly different between patients with self-healed leishmaniasis compared to healthy controls and cases with active CL. Among various differentially expressed miRNAs in patient plasma and *L. braziliensis*-infected THP-1 cells, we selected miR-548d-3p that was upregulated in both settings for further validation.

MiR-548d-3p inhibition in THP-1 cells reduced early parasite growth and increased MCP1/CCL2, RANTES/CCL5, and IP-10/CXCL10, whereas in self-healed patients, MCP1/CCL2, RANTES/CXCL5, and IL-8/CXCL8 were decreased, and MIG/CXCL9 and IP-10/CXCL10 were increased compared with active cases. Collectively, these data suggest that *L. (V.) braziliensis* exploits miRNAs to modulate the production of discrete sets of pro-inflammatory cytokines that are involved in lesion resolution.

## Materials and Methods

### Ethics Statement

The experimental protocols were approved by the ethics committee of the Faculdade de Medicina, Universidade de São Paulo (CAAE 35670314.0.1001.0065) and are in the accordance with the World Medical Association Declaration of Helsinki on Ethical Principles for Medical Research lnvolving Human Subjects of 1964 with latest amendment of 2013. All individuals agreed to participate by signing the Informed Consent Form.

### Patients

Individuals of both gender and age from 15 to 60 years old were selected from endemic areas in Pernambuco state, Northeastern Brazil where *L. (V.) braziliensis* is the predominant species causing CL. Five patients with active disease were chosen based on the presence of up to five cutaneous lesions, confirmed diagnosis of leishmaniasis and absence of local concomitant bacterial infections, comorbidities such as HIV/aids, diabetes mellitus, dermatitis, peripheral vascular diseases, and previous chemotherapy. The diagnosis of active cases was confirmed by submitting sample to direct microscopic parasitological exam of lesion scrapings, by culture, by inoculation into hamsters for parasitological recovery or by polymerase chain reaction specific for *Viannia* subgenus (Brito et al., 2009).

Five self-healed patients with a history of previous cutaneous leishmaniasis were also recruited, showing characteristic scars, confirmed diagnosis, and absence of abovementioned comorbidities, co-infections, and previous chemotherapy. Another five healthy individuals represented the control group, being recruited from non-endemic areas and without previous leishmaniasis, abovementioned comorbidities, or co-infections.

**Figure 1:**
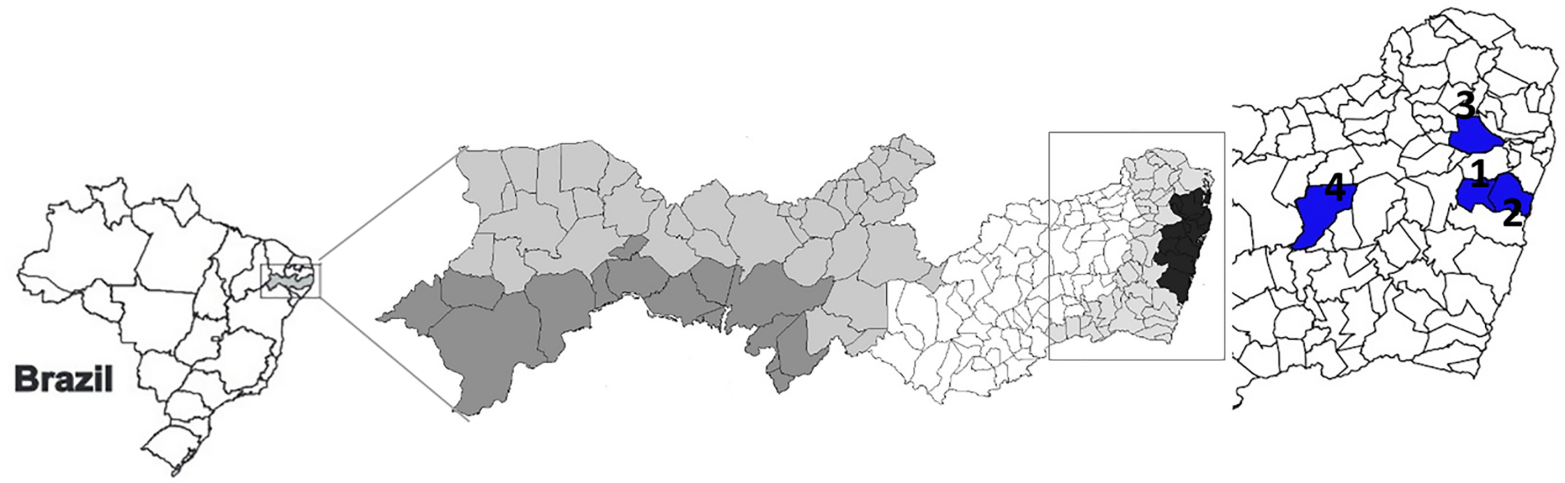
Cartographical representation of the State of Pernambuco, Northeastern Brazil. Regions in blue indicate Moreno (1), Jaboatão (2), Paudalho (3), and Bezerros (4) municipalities.

The patients with active or self-healed leishmaniases were from the municipalities of Paudalho, Moreno, Jaboatão, and Bezerros, localities close by preserved remnants of Atlantic forest, in the State of Pernambuco, Northeast Brazil, where intertwine rural and urban environments where they live and work (**Figure 1**).

After confirmed diagnosis, four milliliters of whole blood were collected in EDTA from each individual, and plasma stored at −80°C until use.

### Parasites

*Leishmania* parasites were previously isolated from a patient with mucosal leishmaniasis at Corte de Pedra, Bahia, Brazil, and characterized as *L. (V.) braziliensis* by the *Leishmania* Collection at Fundação Oswaldo Cruz—CLIOC/FIOCRUZ. To preserve the infectivity, the parasites were inoculated *via* intraperitoneal route and maintained through regular passages in hamster (*Mesocricetus auratus*). Amastigotes were then purified from the spleen of hamster and expanded in axenic culture with Schneider’s insect medium (Sigma-Aldrich, USA) containing 100 UI/ml penicilin and 100 μg/ml streptomycin and supplemented with 10% heat-inactivated fetal calf serum (FCS) (Cultilab, Brazil) at 26°C. The amastigote-derived promastigotes were cryopreserved in aliquots and thawed for use in specific experiments. Promastigotes were cultured in Schneider’s insect medium (Sigma-Aldrich, USA) containing 100 UI/ml penicilin and 100 μg/ml streptomycin and supplemented with 10% heat-inactivated fetal calf serum (FCS) (Cultilab, Brazil) at 26°C. The parasites used in the experiments were at the stationary phase of growth and with no more than four passages in culture.

### Infection of Macrophages With *L. (V.) braziliensis*

THP-1 monocytic cell line (ATCC) was maintained in RPMI 1640 medium (Sigma-Aldrich, USA) supplemented with 2 mM l-glutamine, 1 mM sodium piruvate, 0.2% sodium bicarbonate, and 5% FCS (complete medium). Then 10^6^ cells in 1 ml of RPMI 1640 medium were plated onto 24-well plates (Costar, USA) and incubated in the presence of 20 ng/ml phorbol myristate acetate (PMA; Sigma-Aldrich, USA) for 48 h at 37°C in a humid atmosphere with 5% CO_2_ to allow differentiation into macrophages (Tsuchiya et al., 1982). In experiments for parasite load analysis, round coverslips were placed in the well. Non-adherent cells were then removed and *L. (V.) braziliensis* promastigotes were added to the wells in triplicates (parasite:cell ratio = 5:1) and incubated for 4 h at 33°C in humid atmosphere with 5% CO_2_ to allow infection of macrophages. Non-infected cells were maintained only with culture medium, being the negative control of the experiment. After washing out the non-internalized parasites, complete RPMI medium was added to the wells, beginning the experimental period (0 h). The plates were then maintained for 6 or 24 h at 37°C in a humid atmosphere with 5% CO_2_.

### Evaluation of Parasite Load in Macrophages

Glass coverslips were removed from the wells and stained with panoptic dyes (Instant Newprov, Brazil) and mounted on glass slides for evaluation of parasitism. A total of 900 cells were counted for each experimental condition, 300 cells/coverslip, under light microscope (Carl Zeiss, Germany), and the number of parasites per 100 cells calculated as [(number of parasites/number of infected cells) × (number of infected cells/total number of cells) × 100].

### RNA Extraction, Reverse Transcription, and Pre-Amplification

Total RNA extraction from adherent THP-1 cells was performed using the miRVana PARIS isolation kit (Thermo Fisher, USA), according to the manufacturer’s instructions, and RNA integrity was determined in spectrophotometer as an OD260/280 absorption ratio between 1.8 and 2.1. The total RNA purification in plasma samples was performed using the miRNeasy Serum/Plasma kit (Qiagen, USA), with the addition of a spike-in control (*Caenorhabditis elegans* cel-miR-39) to ensure the quality of the procedure and to allow qPCR normalization, according to the manufacturer’s instructions. Complementary DNA (cDNA) to template RNA purified from THP-1 cells and plasma samples was synthesized with miScript II RT kit (Qiagen, USA). Briefly, 250 ng of total RNA from THP-1 cells were added to 2 μl of 5× miScript HiSpec Buffer, 1 μl of 10× Nucleics Mix, and 1 μl of miScript Reverse Transcriptase Mix. RNase-free water was added to a final volume of 10 μl. The RNA was incubated for 60 min at 37°C to insert poly-A tail downstream of the miRNA sequence and anneal a T-tail tag for the cDNA elongation. The enzyme was inactivated at 95°C for 5 min. The reaction was performed in the Mastercycler Gradient thermal cycler (Eppendorf, Germany), and the product was stored at −20°C until use. The reverse transcription reaction for plasma samples followed the same protocol, with the manufacturer’s instructions to add 4.5 µl of the purified total RNA. Then, 40 µl of DEPC water was added into each 10 µl RT-PCR product and submitted to a pre-amplification reaction (preAmp), using the miScript PreAmp PCR Kit (Qiagen, USA) according to the manufacturer’s instructions. Then the samples were diluted 10× and stored at −20°C.

### Quantitative Real-Time PCR for miRNA

miRNA expression was evaluated with the miScript microRNA PCR array (Qiagen, USA), focusing on inflammation, and auto-immunity pathway-related molecules (MIHS-105Z). Ready-to-use qPCR plates containing a set of 84 specific primers for miRNAs and 12 internal controls were filled in with the previously prepared master mix containing PCR Buffer, SYBR Green, and the 10-fold diluted cDNA for *in vitro* infected THP-1 macrophages or preAmp samples of plasma samples. Quantitative PCR conditions were 40 cycles of 94°C for 15 s, 55°C for 30 s, and 70°C for 30 s. Normalization of miRNA expression in THP-1-derived macrophages was performed using SNORD95 and RNU6-6p as reference genes amplified in the qPCR plate. The relative expression levels were calculated using the Comparative Ct method, with non-infected cells being considered as the calibrator group.

For plasma samples, miRNA expression was also evaluated by relative quantification after previous normalization described by Marabita et al. (2016). The cel-miR-39 spike-in control was considered as a technical reference. Simultaneously, a geometric mean of all expressed miRNAs was used as a normalization factor to calculate relative expression to a calibrator group, which varied depending on the analysis.

### *In Silico* miRNA Target Prediction

Target prediction strategy was performed in two different platforms, considering the miRNAs differentially expressed in the *in vitro* experiment. For an initial screening in human leishmaniasis pathway, we used DIANA-miRpath 3.0 server in the reverse search module (Vlachos et al., 2015), with Targetscan (Agarwal et al., 2015) as the chosen algorithm. To discover potential interactions with other biological pathways related to human leishmaniasis pathogenesis, we performed a second analysis using MiEAA (MiRNA Enrichment Analysis and Annotation), which integrates data from different databases such as miRBase, miRWalk, and miRTarBase (Backes et al., 2016).

### *In Vitro* miRNA Inhibition

The inhibition of miR-548d-3p in THP-1-derived macrophages was performed in an *in vitro* infection experiment through a transient transfection protocol. Assays with three different concentrations (3, 10, and 30 nM) of the mirVana^®^ miR-548d3p inhibitor (Ambion, USA) or mirVana^®^ miRNA Mimic, scramble Negative Control (Ambion, USA) were performed, and 10 nM concentration was chosen for further use (**Figure S1**). At the end of the experiment, the cell viability was evaluated by Trypan blue exclusion test when viability higher than 95% was seen in all conditions. Before the addition of *L. (V.) braziliensis* promastigotes, a solution containing the miR-548d-3p inhibitor or the negative control together with 3 μl of FUGENE transfection reagent (Promega, USA) diluted in 500 μl of RPMI medium previously incubated for 20 min at room temperature was added into each well, and maintained for 24 h. Simultaneously, non-transfected cells received only complete RPMI medium. The experiment continued with promastigote infection for evaluation of parasitism, and chemokine levels in supernatants collected and stored at −80°C until use.

### Evaluation of Chemokine Production

Chemokine quantification in culture supernatants was performed using CBA – Human Chemokine Kit (BD Biosciences, USA) in accordance with manufacturer’s instructions. Briefly, 50 µl of capture beads for MCP1/CCL2, RANTES/CCL5, IL-8/CXCL8, MIG/CXCL9, and IP10/CXCL10, 50 µl of Detection Reagent, and 50 µl of the studied sample or standard were added consecutively to each sample tube and incubated for 3 h at room temperature, in the dark. Next, the samples were washed with 1 ml of Wash buffer, and centrifuged. After discarding the supernatant, the pellet was resuspended in 300 µl buffer and analyzed in a FACS LSR Fortessa flow cytometer (BD Biosciences, USA). Raw data was then analyzed using FCAP Array software (BD Biosciences, USA). The detection limits of each chemokine were as follows: 2.7 pg/ml for MCP1/CCL2, 1.0 pg/ml for RANTES/CCL5, 0.2 pg/ml for IL-8/CXCL8, 2.5 pg/ml for MIG/CXCL9, and 2.8 pg/ml for IP10/CXCL10.

### Statistical Analysis

Regarding *in vitro* miRNA expression, statistical analyses were performed with Qiagen miScript miRNA PCR Array Data Analysis online software, where data were submitted to an integrated Student’s t test under the manufacturer’s recommendation that was applied in the previous similar work (Muxel et al., 2017). *Ex vivo* data were also submitted to Student’s t test, with Bonferroni’s correction, using Microsoft Excel 365. Parasite load data were analyzed by ANOVA with Tukey’s post-test, and data from chemokine quantification by Kruskal-Wallis test with Bonferroni’s correction. The differences were considered significant when *P* < 0.05.

## Results

### miRNA Expression in *L. (V.) braziliensis* Promastigote-Infected THP-1 Cells

**Figure 2:**
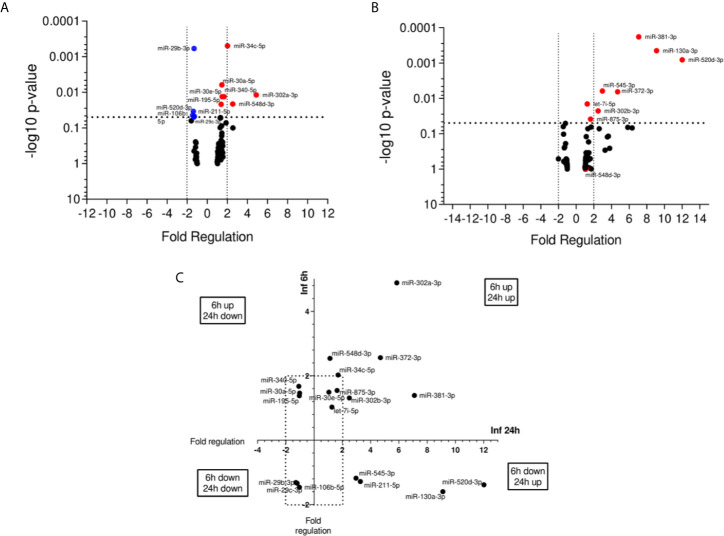
miRNA profiles of *L. braziliensis* infected THP-1-macrophages. Volcano plot of differential expression of miRNA in *L. (V.) braziliensis* promastigote-infected THP-1 macrophages in relation to non-infected cells at 6 h **(A)** and 24 h **(B)** post-infection compared to uninfected-macrophages. Each dot represents one miRNA. Red dots indicate the up-regulated miRNAs, and the blue dots represent the down-regulated miRNAs (P < 0.05). Black dotted line corresponds to p = 0.05, log 10. The relative up- and down-regulation of miRNAs, expressed as boundaries of 2 or -2 of Fold Regulation, respectively. P-value was determined based on two-tailed Student’s t test. Significantly expressed miRNAs in different times distributed in four groups **(C)**. Experiments were repeated three times.

We evaluated miRNA expression at 6 and 24 h post infection with *L. (V.) braziliensis* promastigotes, considering non-infected THP-1 cells as the calibrator group. 19 out of 84 miRNAs presented significant alteration in expression (P < 0.05). At 6 h p.i., seven miRNAs were upregulated while five were down-regulated (**Figure 2A**). In contrast, eight miRNAs were up regulated in 24 h (**Figure 2B**). From these results, we observed that these miRNAs expression was modulated through time and classified them into four groups. MiR-106b-5p, miR-29b-3p, and miR-29c-3p were the down-regulated molecules at both 6 and 24 h, while miR-195-5p, miR-30a-5p, and miR-340-5p were up-regulated at 6 h and down-regulated at 24 h. There were also down-regulated miRNAs at 6 h and upregulated at 24 h, such as miR-130a-3p, miR-211-5p, miR-520d-3p, and miR-545-3p, whereas let7i-5p, miR-30e-5p, miR-302a-3p, miR-302b-3p, miR-34c-5p, miR-372-3p, miR-381-3p, miR-548d-3p, and miR-875-5p were upregulated at both time-points (**Figure 2C**).

### miRNA Profiling of Serum From ATL Patients

The participants of this study were predominantly male and the active disease patients were younger (mean 20 and median 20 years old, minimum-maximum:18-22 ya) than self-healed patients (mean 26.2 and median 27 years old, minimum-maximum: 21-30 ya). The number of present or past lesions, and their localization were similar in both groups. The patients with active disease presented lesions characteristic of the localized form of CL (rounded, ulcerated, with well-defined and elevated edges and granulomatous bottom) on uncovered parts of the body, with evolution ranging from 15 to 28 days. The self-healed individuals presented typical scars without previous anti-*Leishmania* chemotherapy, with healing time ranging from three to nine months (**Table 1**).

**Table 1:** Demographic and clinical data of active disease and self-healed patients.

	Code	Gender/Age (years old)	Occupation	Locality	Clinical form	Lesions (n)	Size (cm)	Evolution	Lesion site	Parasite search	PCR	Isolation Parasite	IDRM
**Active disease patients**	0001	M/19	Military	Paudalho/PE	Ulcerated	01	1.0 × 2.0	15 days	Left leg	+	+	ND	+
	0002	M/21	Military	Paudalho/PE	Ulcerated	01	0.5 × 0.5	15 days	Right flank	+	+	–	+
	0003	M/22	Military	Paudalho/PE	Ulcerated	01	0.5 × 0.5	15 days	Right hand	+	–	+	+
	0004	M/20	Military	Paudalho/PE	Ulcerated	01	0.6 × 0.6	1 month	Right hand	+	–	–	+
	0005	M/18	Student	Moreno/PE	Ulcerated	01	1.5 × 1.5	15 days	Left Leg	+	+	+	+
	**Code**	**Gender/Age**	**Occupation**	**Provenance**	**Reported clinical form**	**Scars (n)**	**Size (cm)**	**Healing time**	**Scar sites**	**Parasite search**	**PCR**	**Parasite Isolation**	**IDRM**
**Self-healed patients**	0006	M/27	Farmer	Moreno/PE	Ulcerated	02	2.0 × 3.0; 1.0 × 1.0	4 months	Right foot	+	–	–	ND
	0007	M/29	Machine operator	Jaboatão/PE	Ulcerated	01	4.0 × 3.0	9 months	Left leg	−	+	–	ND
	0008	M/30	Engineer	Bezerros/PE	Ulcerated	02	2.0 × 2.0	6 months	Left leg	−	+	–	ND
	0009	F/24	Housewife	Moreno/PE	Ulcerated	01	1.5 × 1.5	4 months	Right leg	+	+	–	ND
	0010	M/21	Farmer	Moreno/PE	Ulcerated	01	2.0 × 2.0	3 months	Right thigh	−	+	+	ND

M, male; F, female; L, left; R, right; PCR, polymerase chain reaction; IDRM, Montenegro skin test; ND, not done.

**Figure 3:**
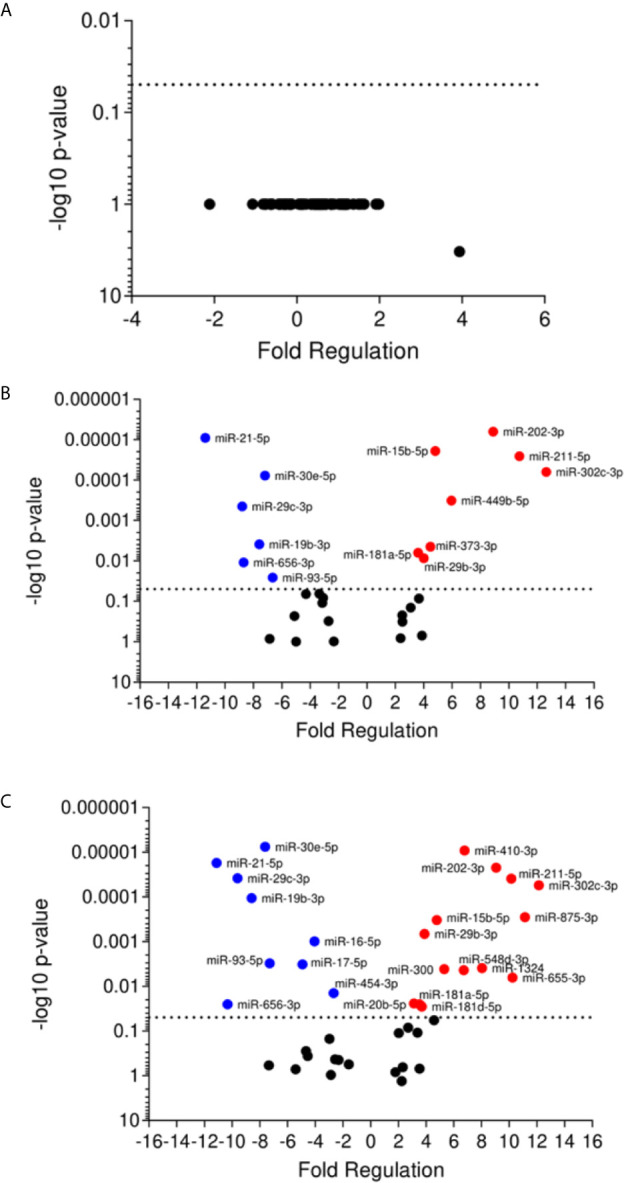
Volcano plot of differential expression of miRNA in plasma of cutaneous leishmaniasis patients. Volcano plot of differential expression of miRNA in plasma samples of active disease patients **(A)** and self-healed **(B)** compared to healthy individuals, and self-healed **(C)** compared to active disease patients. Each dot represents one miRNA. Red dots indicate the up-regulated miRNAs, and the blue dots represent the down-regulated miRNAs (P < 0.05). Black dotted line corresponds to p=0.05, log 10. The relative up- and down-regulation of miRNAs, expressed as boundaries of 2 or -2 of Fold Regulation, respectively. P-value was determined based on two-tailed Student’s t test. P < 0.05 (Student *t* test and Bonferroni correction).

The miRNA expression in plasma samples of patients with active disease was not significantly different compared to the control group (**Figure 3A**). In contrast, self-healed individuals presented 14 differentially expressed miRNAs, eight of them up-regulated (miR-15b-3p, miR-29b-3p, miR-181-5p, miR-202-3p, miR-211-5p, miR-302c-5p, miR-373-3p, miR-449-5p), and six down-regulated (miR-19b-3p, miR-21-5p, miR-29c-3p, miR-30e-5p, miR-656-3p, miR-93-5p) in relation to the control group (**Figure 3B**). When comparing self-healed patients with the active disease group, we found a total of 23 significantly altered miRNAs, with 14 of these up-regulated (miR-15b-5p, miR-20b-5p, miR-29b-3p, miR-181a-5p, miR-181d-3p, miR-202-3p, miR-211-5p, miR-300, 302c-5p, miR-410-3p, miR-548d-3p, miR-875-3p, miR-655-3p, miR-1324), and nine down-regulated (miR-16-5p, miR-17-5p, miR-19b-3p, miR-21-5p, miR-29c-3p, miR-30e-5p, miR-93-5p, miR-454-3p, miR-656-3p) (**Figure 3C**). Our data suggest distinct miRNA profiles in plasma samples of active and self-healed ATL patients.

### Concomitant Altered Expression of miRNAs in *In Vitro* and *Ex Vivo* Experiments

To focus on understanding the miRNA modulation and function during infection, we searched for correlations between up- and down-regulated miRNAs. We found some miRNAs differentially expressed in infected THP-1 cells and plasma samples of ATL patients (**Table 2**). The miR-548d-3p and miR-875 were upregulated in self-healed patients and *in vitro* at 6 and 24 h of incubation post-infection. Despite that, miR-211-5p and miR-29b-3p were upregulated in self-healed patients, but down-regulated at 6 h of incubation post-infection *in vitro*. Down-regulated expression was observed for miR-29c-3p *ex vivo*, showing similar modulation in the *in vitro* experiment at both time points. Finally, miR-30e-5p was upregulated *in vitro* at 6 and 24 h of incubation post-infection and down-regulated in plasma samples (**Figures 2C** and **3C**).

**Table 2:** Set of miRNAs significantly expressed both in *in vitro* and *ex vivo* contexts.

Up regulated	Down regulated
miR-410-3p	**miR-30e-5p**
miR-202-3p	miR-21-5p
miR-211-5p	**miR-29c-3p**
miR-302c-3p	miR-19b-3p
miR-15b-5p	miR-16-5p
**miR-875-3p**	miR-17-5p
**miR-29b-3p**	miR-93-5p
**miR-548d-3p**	miR-454-3p
miR-300	miR-656-3p
miR-1324	
miR-655-3p	
miR-181a-5p	
miR-181d-5p	
miR-20b-5p	

miRNAs in bold, molecules modulated in similar ways in both experiments.

### miRNA Predicted Targets and Interactions With Biological Pathways Related to ATL Pathogenesis

**Figure 4:**
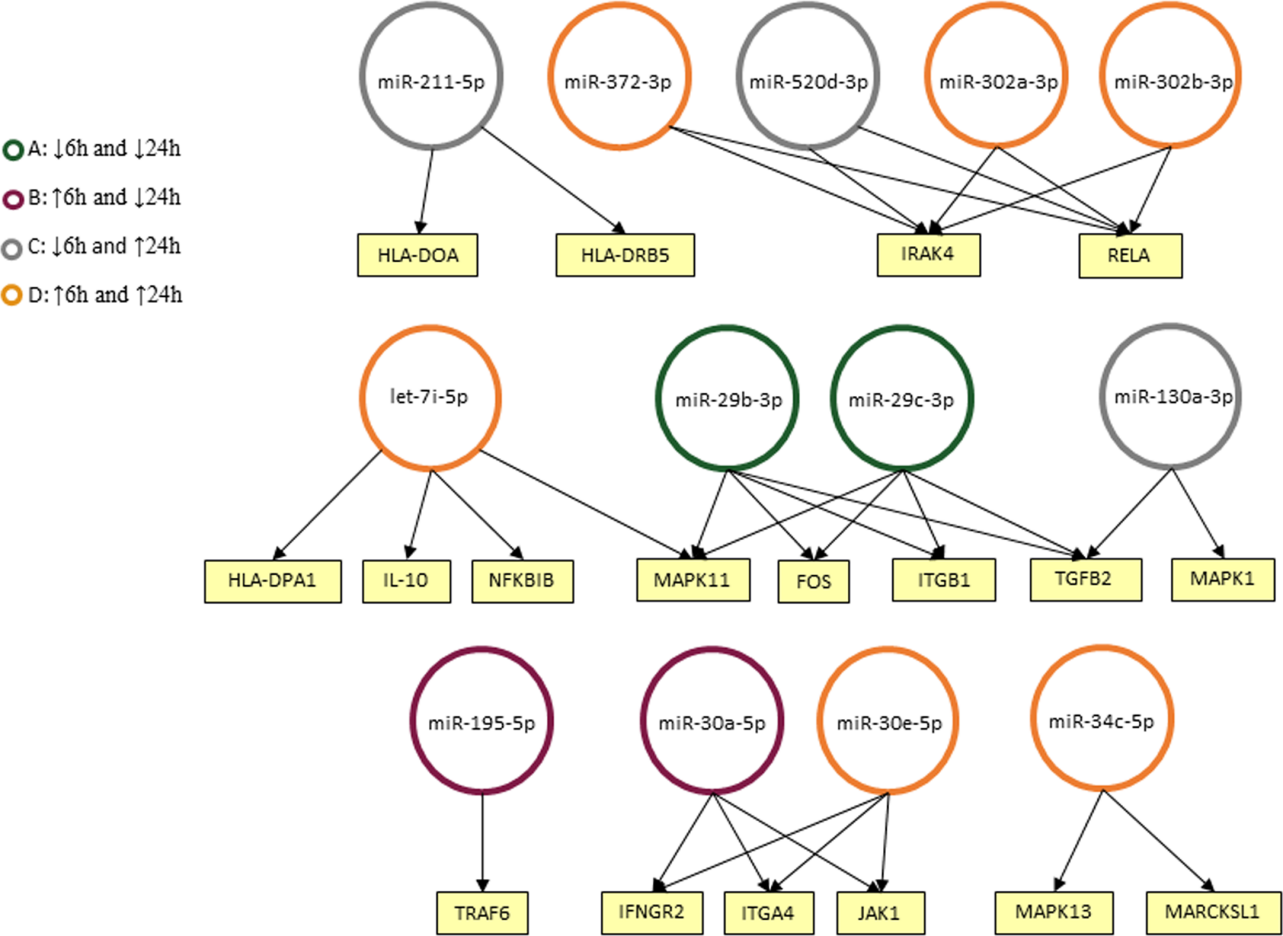
Predicted interactions between the set of differentially expressed microRNAs in THP-1 cells after 6 and 24 h post infection. The genes on which they are suggested to act in the Leishmaniasis pathway in humans are shown as seen in KEGG. miRNAs were classified into four groups according to their modulation through time: down-regulated in 6 and 24 h **(A)**, up-regulated on 6 h and down-regulated in 24 h **(B)**, down-regulated in 6 h and up-regulated in 24 h **(C)** and up-regulated in both timepoints **(D)**.

We used Diana MiRPath 3.0 with TargetScan2 as the chosen algorithm to predict miRNA/mRNA interactions, focusing on miRNAs modulated in infected THP-1 cells. Among the interactions predicted in the initial analysis using the Diana platform, there were cytokines encoded by the *TGFB2* and *IL10* genes, MHC class II proteins (HLA-DPA1, HLA-DRB5, and HLA-DOA) and genes related to signaling pathways (e.g. MAPK1, MAP3K7, IRAK4) (**Figure 4**).

**Figure 5:**
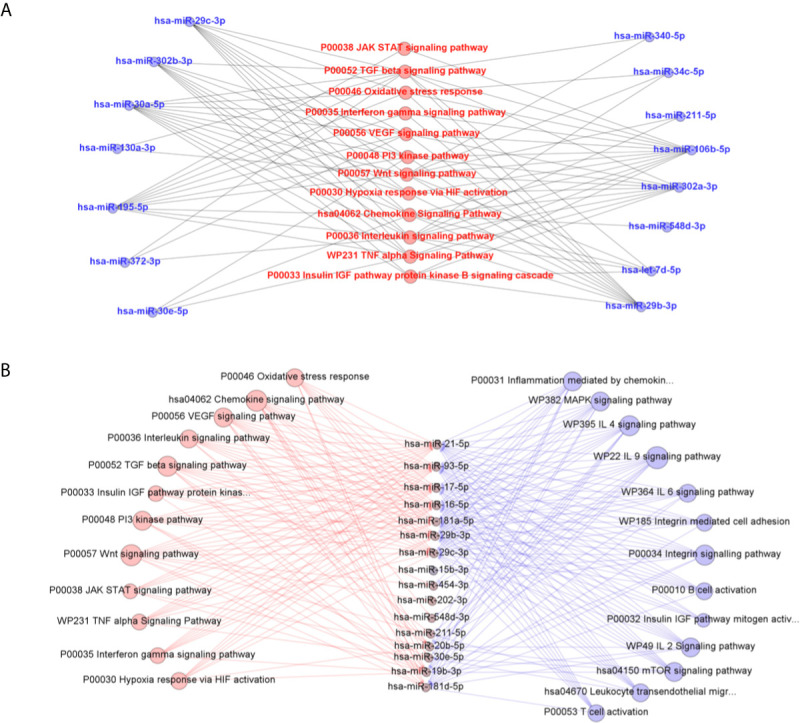
Predicted interactions between the set of differentially expressed microRNAs in THP-1 cells after 6 and 24 h post infection with *L. braziliensis* **(A)** and in plasma samples from self-healed patients compared to active disease individuals **(B)** and the biological pathways related to inflammatory response on which they are suggested to act according to MiEAA algorithms. In **(B)**, pathways in red were predictably targeted by *in vitro* and *ex vivo* miRNA sets, while the others in blue were evidenced only in miRNAs significantly quantified in plasma.

Further predictions made in MiEAA platform showed, in more than one classification system (PANTHERDB, WikiPathways, and KEGG), some pathways known to be important in the parasite-host interaction that can be regulated by the expressed microRNAs in *L. braziliensis*-infected THP-1 cells (**Figure 5A**) and in self-cured patients plasma compared with active patient sample (**Figure 5B**). Cytokine signaling pathways, such as IFN-γ, TNF-α, and TGF-β, are known to be involved in the immune response against *Leishmania*. Also, signal transduction pathways such as JAK-STAT and PI3K were putative targets of differentially expressed miRNAs, as well as the VEGF, Wnt, and HIF-1 pathways. There is also a potential interference of miRNAs expressed in the oxidative stress pathway. Finally, the signaling cascade activated by IGF receptors may be influenced by the differentially expressed microRNAs. Searching predicted pathways targeted by the circulating microRNAs present in plasma, we observed the inflammation mediated by chemokines and cytokines and the chemokine signaling pathways. Besides, important pathways involved in B cell development like B cell activation and mTOR signaling pathways were predicted. Pathways involved in Th17 and Th2 differentiation and T cell proliferation such as T cell activation, IL-4, IL-6, and IL-2 signaling were evidenced.

Among various differentially expressed miRNAs, we selected miR-548d-3p that was upregulated in patient plasma and *L. braziliensis*-infected THP-1 cells and targets only two pathways for further validation.

### Effect of miR-548d-3p Inhibition on Parasite Load in THP-1 Infection With *L. (V.) braziliensis*

**Figure 6:**
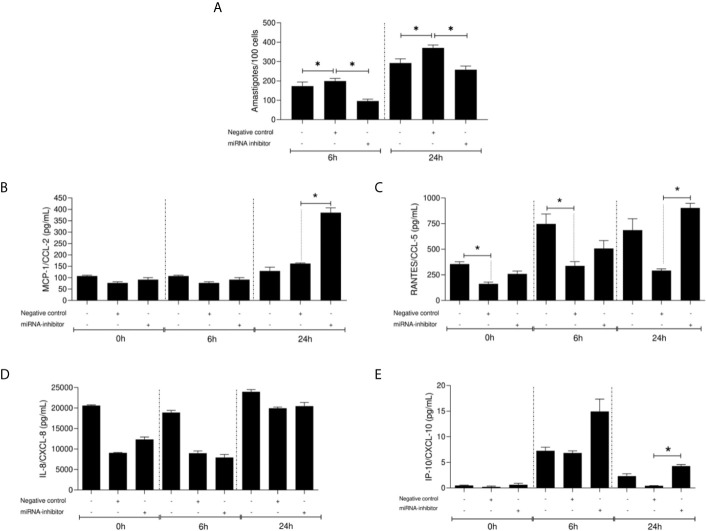
Parasite load (number of amastigotes/100 cells) **(A)** and chemokine levels (pg/ml) **(B–E)** in *L. (V.) braziliensis* promastigote-infected THP-1 cells transiently transfected with miR-548d-3p inhibitor at 6 and 24 h post-infection. One experiment was carried out by adding the miR-548d-3p inhibitor (10 nM) or negative control (scrambled miRNA; 10 nM) with the transfection reagent diluted in RPMI medium or only RPMI medium (non-transfected cells) to wells containing 10^6^ THP-1 adherent cells and maintained for 24 h at 37°C (5% CO2) then infected with *L. (V.) braziliensis* promastigotes. **(A)** * = P < 0.05 (one way ANOVA and Student *t* test). MCP1/CCL2 **(B)**, RANTES/CXCL5 **(C)**, IL-8/CXCL8 **(D)**, and IP-10/CXCL10 **(E)** concentrations were measured by flow-cytometry using the CBA kit. **(B–E)** * = p <0.05 (Kruskal-Wallis and Bonferroni tests).

The function of miR548d-3p during *L. braziliensis* infection was evaluated using 10 nM specific inhibitor or scrambled miRNA. At both 6 and 24 h post-infection, a significant decrease was observed in parasite load when miR-548d-3p was inhibited (P < 0.05), compared to transfection with scrambled RNA, negative control (**Figure 6A**).

### miR-548d-3p Inhibition on Chemokine Production in *L. (V.) braziliensis*–Infected THP-1 Cells

Inhibition of miR-548d-3p did not affect the production of CCL2 by infected THP-1 cells at 6 h p.i. but led to a >2-fold increase in secretion of CCL2 at 24 h p/i/compared to both untreated infected cells and cells treated with the scrambled RNA-negative control (**Figure 6B**). In contrast, CCL5 production appeared more susceptible to modulation by transfection of the scrambled control RNA and use of the inhibitor tended to normalize the production to that seen in untransfected cells (**Figure 6C**). The production of CXCL8 and CXCL10 were not significantly affected by the miR-548d-3p inhibitor in comparison to untransfected cells but a small but significant increase in CXCL10 was observed compared to the scrambled inhibitor at 24 h p.i. (**Figures 6D, E**).

### Chemokine Levels in Plasma of ATL Patients

**Figure 7:**
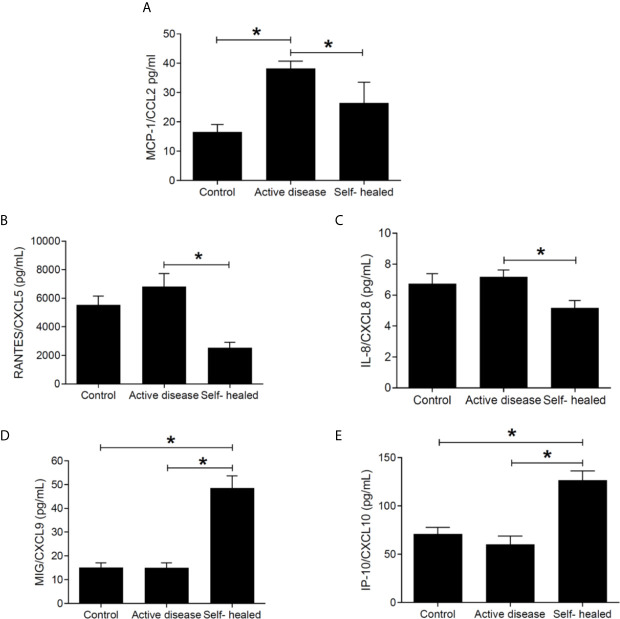
Chemokine concentration (pg/ml) in plasma of cutaneous leishmaniasis patients and controls. MCP1/CCL2 **(A)**, RANTES/CXCL5 **(B)**, IL-8/CXCL8 **(C)**, MIG/CXCL9 **(D)**, and IP-10/CXCL10 **(E)** concentrations were measured by flow-cytometry using the CBA kit. * = p < 0.05 (Kruskal-Wallis and Bonferroni tests). N =5 patients and controls per group.

CCL2 was found at significantly higher concentration in plasma samples of patients with active disease compared to self-healed and control groups (**Figure 7A**), whereas CXCL5 and CXCL8 were decreased in self-healed cases compared to active cases (**Figures 7B, C**). Significantly higher concentrations of CXCL9 (**Figure 7D**) and IP-10 (**Figure 7E**) were seen in self-healed patients in relation to healthy individuals and patients with active disease (P < 0.05).

## Discussion

Macrophages exert a dual role in the pathogenesis of CL, being both host cell and also the main effector cell for parasite clearance (Tomiotto-Pellissier et al., 2018). The disease outcome depends on the interplay between *Leishmania* and the host immune responses which govern these opposing macrophage functions. *Leishmania* employ strategies to evade the host immune response, including altering the miRNAs expression (Lemaire et al., 2013; Muxel et al., 2017; Muxel et al., 2018; Nunes et al., 2018; Fernandes et al., 2019; Paul et al., 2020). In this context, having access to *L. braziliensis*-infected active and self-healed CL patients, we searched for differentially expressed miRNAs in plasma and in parallel conducted an *in vitro* study using *L. (V.) braziliensis* infected-human monocyte-derived THP-1 cells. With this approach, we sought to attribute altered miRNA profiles to mechanisms of disease pathogenesis.

Alterations of miRNA expression were seen in self-healed patient samples compared with active cutaneous leishmaniasis cases and healthy controls, the latter being indistinguishable by miRNA profile. Thus, these data suggest that host cells from individuals that cure without treatment are more active in altering miRNA expression upon *L. (V.) braziliensis* infection, although we cannot rule out at this preliminary stage in our investigation whether this is confounded by other factors unrelated to infection e.g., environmental exposure or host genetics. Concerning environment, the patients were from endemic areas, from nearby cities with similar climate and environmental characteristics. Differentially expressed miRNAs have been related to inflammatory chemokine levels, and this may contribute to the self-healing nature of these patients. An additional weakness of the study is that parasites from these patients were not genotyped or functionally evaluated. This may be important given that some strains of *L. braziliensis* are more susceptible to oxidative stress than others, and induce lesions with a higher tendency to spontaneous healing (Souza et al., 2010; Sarkar et al., 2012). In addition, we have to consider a proper balance between regulatory and pro-inflammatory mediators, especially IFN-γ and IL-10, that may differ depending on the hosts background, important for the lesion to heal (Gomes-Silva et al., 2007; de Assis Souza et al., 2013).

Evaluation of altered miRNA expression *in vitro L. (V.) braziliensis* infected-human monocyte-derived THP-1 cells showed a set of miRNAs also found altered in plasma of leishmaniasis patients. In silico prediction of THP-1 expressed miRNA targets and interactions with biological pathways suggested a link between the differentially expressed miRNAs and altered expression targeted cytokine, chemokine, and signaling pathways, signal transduction pathways, and others.

A total of 19 out of 84 miRNAs exhibited an altered *in vitro* expression compared with non-infected THP-1 cells either at 6 or 24 h of incubation after infection, showing that *Leishmania* can modulate these molecules in a temporally distinct manner during the early stages of *in vitro* infection as seen by others (Guerfali et al., 2008; Bazzoni et al., 2009; Lemaire et al., 2013). In the in-silico predictions using DIANA miRPath 3.0, considering *Leishmania* infection pathway, upregulated miR-195-5p at 6 h may target tumor-necrosis factor receptor-associated factor 6 (TRAF6), an important player in signal transduction of both the TNF receptor (TNFR) superfamily and the interleukin-1 receptor (IL-1R). These are crucial to ultimately activate transcription factors, such as nuclear factor kappa B (NF-κB) and interferon-regulatory factor (IRF), to induce immune and inflammatory responses (Ye et al., 2002; Wang et al., 2010). In addition, two isoforms of miR-30 family, miR-30a-5p, and miR-30e-5p, were suggested to target Interferon gamma receptor 2 (IFNGR2), Janus kinase 1 (JAK1), Integrin subunit alpha 4 (ITGA4) genes throughout time. These predicted interactions suggest participation in parasite control mechanism and inflammatory process. Other important events of the immune response such as Toll-like receptor signaling and antigen presentation were also predicted to be compromised by the influence of let-7i-5p, miR-130a-3p, miR-520d-3p, and two isoforms of miR-302.

Other relevant pathways that are known to play a role in the adaptive immune response in cutaneous leishmaniasis were targeted by miRNAs identified exclusively in plasma samples from self-healed patients compared with active disease subjects. Pathways related to T and B cell activation including mTOR pathway that can modulate B cell development (Limon and Fruman, 2012; Iwata et al., 2017) were predicted. Cytokine related pathways such as IL-2, IL-4, IL-6, and IL-9 were also evidenced. These different pathways potentially targeted by circulating microRNAs might reflect the diversity of cells participating in the immune response in humans, in contrast to exclusively monocyte/macrophage *in vitro* experiment.

Among altered miRNAs in the present study, other miRNAs belonging to the same family were previously analyzed in *Leishmania* infection. The modulation of miR-29b, miR-29c, and miR-30e were observed in *L. amazonensis-*infected murine macrophages (Muxel et al., 2017; Muxel et al., 2018; Fernandes et al., 2019). Upregulation of miR-29b was observed in *L. major*-infected human monocyte-derived macrophages, whereas this miRNA was down-regulated in *L. donovani* infection (Geraci et al., 2015). Stimulation of the nucleotide-binding oligomerization domain containing 2 (NOD2) induces miR-29 family upregulation, resulting in downregulation of IL-12p40 without alteration to IL-6, TGF-β or IL-10 production (Yu et al., 2002; Guo et al., 2012; Reveneau et al., 2012). However, NOD2-stimulation increases the levels of IL-1β, IL-6, and IL-23 cytokines, Nitric Oxide Synthase 2 (NOS2) expression and nitric oxide (NO) production during *Leishmania* infection (Lima-Junior et al., 2013).

We also searched for other biological pathways that could be affected during *Leishmania* infection, and our predictions using the MiEAA platform pointed to some involved in inflammation and wound healing as follows. Also, our predictions highlighted TNF, IFN-γ, and TGF-β signaling pathways, cytokines with respective proinflammatory and regulatory roles in *Leishmania* infection (Souza et al., 2012; de Assis Souza et al., 2013; Souza et al., 2016). The oxidative stress response pathway was also revealed once reactive oxygen and nitrogen species (ROS and RNS) produced during an inflammatory response are an important part of host-defense strategies of organisms to kill the parasite (Kocyigit et al., 2005).

Many characteristics of leishmanial lesions such as microcirculation impairment, metabolic demand for leukocytes, parasite proliferation, and secondary bacterial infection are indicators of a hypoxic event in those lesions (Fraga et al., 2012). Related to this condition, changes in miRNAs that regulate Hypoxia-inducible factor 1 *(*HIF-1) activation in response to hypoxia were also identified in silico. Other possible consequence of a hypoxic, inflammatory microenvironment is the induction of vascular remodeling *via* Vascular endothelial growth factor A/Vascular endothelial growth factor receptor (VEGF-A/VEGFR) expression by HIF-1 influence, which are elevated in the skin of humans and mice infected with *Leishmania* parasites (Fraga et al., 2012; Araujo and Giorgio, 2015). Differentially expressed miRNA affecting VEGF were also observed in our data. Our in-silico predictions also showed that some of the altered miRNAs targets the IGF-I signaling pathway. The role of this hormone in *Leishmania* infection has been long studied with pleiotropic effect in innate and adaptive immune response and pathogenesis in leishmaniases (Reis et al., 2021).

Cutaneous lesions are characterized by chronic inflammation where concur activated CD69^+^ T cells (Diaz et al., 2002), regulatory CD4^+^CD25^+^FOXP3^+^ IL-10–producing T cells, granzyme A CD8^+^ cytotoxic T cells, CD4^+^ IFN-γ–producing T cells (Bourreau et al., 2009; Faria et al., 2009) and where higher expression of gene sets related to the cytolytic pathway is observed (Amorim et al., 2019). The influx of cells into the lesion reflects the role of chemokines and one of miRNA seen altered *in vitro* and patients’ plasma in the present study was miR-548d-3p. miR-548d-3p and others from the same family were reported related to wound healing and inflammation in rheumatoid arthritis and *Leishmania donovani* infection (Wang et al., 2018; Huang et al., 2020) thus we proceeded with functional validation of the miR-548d-3p in *L. braziliensis* infected-THP-1 cells. The miR-548 family is a larger and poorly conserved, encompassing 69 human miR-548 genes located in almost all human chromosomes (Liang et al., 2012). Previous studies showed that miR-548d are processed from the same encoded hairpin cluster of miR-548aa1 (GenBank ID 100500863) and that miR-548d-3p belongs to the cluster family of hsa-miR-548-d1 (miRbase ID MI0003668) (Cummins et al., 2006; Landgraf et al., 2007) transcribed from negative strand of intronic region of ATPase family AAA domain containing 2 (ATAD2, gene ID NM_014109.4) gene located into chromosome 8 (search in miRIAD toll) (Cummins et al., 2006). The transcription of miR-548d1 is related to transcription of the ATAD2 gene, as observed upon glucocorticoid stimulation (Rainer et al., 2009). This information showed the complex changes in miRNA/miRtron expression regulation upon distinct stimuli. ATAD2 has a ATP-binding site and ATPase activity, regulating the assembly of protein complexes (Morozumi et al., 2016), as CREB-binding promoter region or regulating histone hyperacetylation (Koo et al., 2016; Lazarchuk et al., 2020), suggesting the ATAD2/miR-548d can alter gene transcription during infection. ATAD2 inhibits the expression of vascular endothelial growth factor A (VEGFA) by altering miR-520a levels (Hong et al., 2018), linking miR-548d expression to modulation of other miRNAs. Also, ATAD2 can be a target of miRNAs, including miRNAs modulated during *Leishmania* infection including molecules described in our study such as miR-302, miR-373, and miR-93 (Bragato et al., 2018; Fernandes et al., 2019; Kumar et al., 2020).

miR-548d-3p was shown to enhance cell proliferation and inhibit apoptosis in breast cancer cells (Song et al., 2016), suggesting a possible role in inhibition of apoptosis seen in *L. donovani*-infected macrophages (Moore and Matlashewski, 1994). The miR-548 family can regulate expression of High mobility group box1 (HMBG1) a non-histone nuclear protein, a potent stimulator of tissue damage and inflammation through expression of pro-inflammatory cytokines (Martinotti et al., 2015; Son et al., 2019). The miR-548d-3p was seen previously in healing and inflammatory processes. In post-burn wound healing, the vascular endothelial growth factor-A (VEGFA) a key factor involved in the wound healing process was shown to likely be targeted by miR-548d-3p (Huang et al., 2020). In rheumatoid arthritis, an autoimmune inflammatory disease, another member of the miR-548 family, miR-548a-3p, was significantly down-regulated in serum samples targeting Toll-like receptor 4/nuclear factor kappa B (TLR4/NF-kappaB) signaling pathway (Wang et al., 2018). In THP-1 cells infected with promastigotes isolates from post-kala-azar dermal leishmaniasis, other members of the 548-miRNA family, miR-548at-5p, miR-548t-3p, were upregulated when compared to THP-1 cells infected with promastigotes isolated from visceral leishmaniasis patients (Kumar et al., 2020).

Importantly, miR-548d-3p was induced in both self-healed leishmaniasis patient samples and *in vitro L. braziliensis*-infected THP-1 cells. Because the miR-548-3p was found in patients’ plasma, it is likely that it is secreted by *L. braziliensis*-infected THP-1 cells, an aspect deserving further studies. It is known that THP-1 cell line can actively secrete microvesicles and exosomes that may contain miRNAs, such as miR-150 (Zhang et al., 2010) and miR‐103‐3p (Chen et al., 2020). Further, the content of microvesicles and exosomes may be modified by inflammatory conditions, infections including *Leishmania*, apoptosis, etc (Silverman et al., 2010; Baxter et al., 2019; Yao et al., 2019).

Inhibiting miR-548d-3p in THP-1 cells we observed a decrease in parasite load, and an increase in the production of MCP1/CCL2, RANTES/CCL5, and IP-10/CXCL10. In parallel, in plasma of self-healed patients, MCP1/CCL2, RANTES/CCL5, and IL-8/CXCL8 were decreased but increased MIG/CXCL9 and IP-10/CXCL10. We should be cautious to relate the *in vitro* experimental data to the evaluation in plasma. However, we observe a dichotomy impact of miR-548d, when upregulated in the early stage of *in vitro* infection of THP-1 derived monocytes by *L. braziliensis* (6–24 h) that is apparently reducing MCP-1 and RANTES at the infection site, contributing to the control of local inflammatory response, but at the same time, it is enabling parasite growth subverting the inflammatory response and lesion wound healing. These findings, considering the possibility of secretion of miR548d-3p by macrophages, are in line with the upregulated miR548d-3p found in the self-healed plasma patients that may reduce the MCP-1 and RANTES at systemic levels, contributing positively to wound healing modulating the inflammation. High IP-10 and MIG secretion in self-healed patients suggests that the miR-548-3p is not able to control the secretion of these cytokines. Previously, we observed higher levels of IP-10 and MIG, IFN-γ, and TNF in active and self-healed cutaneous leishmaniasis regulating parasite growth control (Souza et al., 2012; de Assis Souza et al., 2013).

Other studies have reported the role of these chemokines in cutaneous leishmaniasis. RANTES/CCL5, together with KC/CXCL1 and MIP-2/CXCL2 (Ohmori and Hamilton, 1994; Lebovic et al., 2001) participate in neutrophil, monocyte, and lymphocyte recruitment to inflammatory focus and interfere in the persistence of cutaneous leishmaniasis lesions (Teixeira et al., 2005; Costa-Silva et al., 2014). In experimental cutaneous leishmaniasis, the upregulation of miR-294 regulated *Ccl*2/*Mcp*-1 mRNA levels and infectivity in *L. amazonensis* infected BALB/c bone marrow-derived macrophages (Fernandes et al., 2019). Similarly, the downregulation of chemokines CCL2, CCL5, CXCL10, CXCL11, and CXCL12 was seen with upregulation of let-7a, miR-25, miR-26a, miR-132, miR-140, miR-146a, and miR-155 in *L. major*-infected human macrophages (Guerfali et al., 2008).

miRNAs are promising tools for diagnosis, treatment, and prognostic markers. Product for diagnosis is a reality mainly for cancers. No miRNA-based therapeutic formulations like miRNA mimics and antagomirs have reached the pharmaceutical breakthrough, but some are currently in clinical trials. In CL caused by *L. braziliensis*, miR-361-3p was appointed as a prognostic marker related to therapeutic failure. The miR-548d-3p evaluated in the present study was shown to exert tumor-suppressive effects in osteosarcoma cells and proposed as a therapeutic tool for osteosarcoma (Chen et al., 2019). Based on our findings, further studies are warranted to more clearly establish a role for miR-548d-3p as a prognostic marker and therapeutic target in cutaneous leishmaniasis.

## Data Availability Statement

The original contributions presented in the study are included in the article/**Supplementary Material**. Further inquiries can be directed to the corresponding author.

## Ethics Statement

The studies involving human participants were reviewed and approved by Comite de Etica e Pesquisa da Faculdade de Medicina da Universidade de São Paulo. The patients/participants provided their written informed consent to participate in this study.

## Author Contributions

HG: Conceptualization, study design, project and researcher supervision, manuscript preparation. MS, and ER-S: Conceptualization, study design, experimental work, data analysis, manuscript preparation. LF-W: study design, researcher supervision, manuscript preparation. SM: study design, experimental work, data analysis, manuscript preparation. LR: experimental work, manuscript preparation. RZ: experimental work. VP and MB: coordination of sample and data collection in endemic area, data interpretation. DL and PK: data analysis, manuscript preparation. All authors contributed to the article and approved the submitted version.

## Funding

This work was supported by the Fundação de Amparo à Pesquisa do Estado de São Paulo (grants 2018/23512-0, 2018/14398-0, and 2018/24693-9, fellowship 2014/14756-2 to MS and 2019/25393-1 to LR), Medical Research Council (grants MR/P024661/1 and MR/S019472), the Conselho Nacional de Pesquisa (research fellowship to HG), the Coordenação de Aperfeiçoamento de Pessoal de Nível Superior (CAPES; fellowship to MS) and LIM 38 (Hospital das Clínicas, Faculdade de Medicina, Universidade de São Paulo).

## Conflict of Interest

The authors declare that the research was conducted in the absence of any commercial or financial relationships that could be construed as a potential conflict of interest.

## Supplementary Material

Supplementary Figure 1 Effect of different concentrations of miR-548d-3p inhibitor or negative control (scrambled miRNA) transiently transfected on parasite load (number of amastigotes/100 cells) in *L. (V.) braziliensis* promastigote-infected THP-1 cells at 6 h **(A)** and 24 h **(B)** post-infection. The experiment was carried out by adding the synthetic molecules at 3nM, 10nM or 30nM with the transfection reagent diluted in RPMI medium or only RPMI medium (non-transfected cells) to wells containing 10^6^ THP-1 adherent cells and maintained for 24 h at 37°C (5% CO_2_) then infected with *L. (V.) braziliensis* promastigotes. **(A)** * = P < 0.05 (one way ANOVA and student t test).
