# Supplementary figures and images for: miR-548d-3p Alters Parasite Growth and Inflammation in Leishmania (Viannia) braziliensis Infection
Source: Front Cell Infect Microbiol. 2021 Jun 10;11:687647. doi: 10.3389/fcimb.2021.687647 (PMC8224172; doi:10.3389/fcimb.2021.687647)

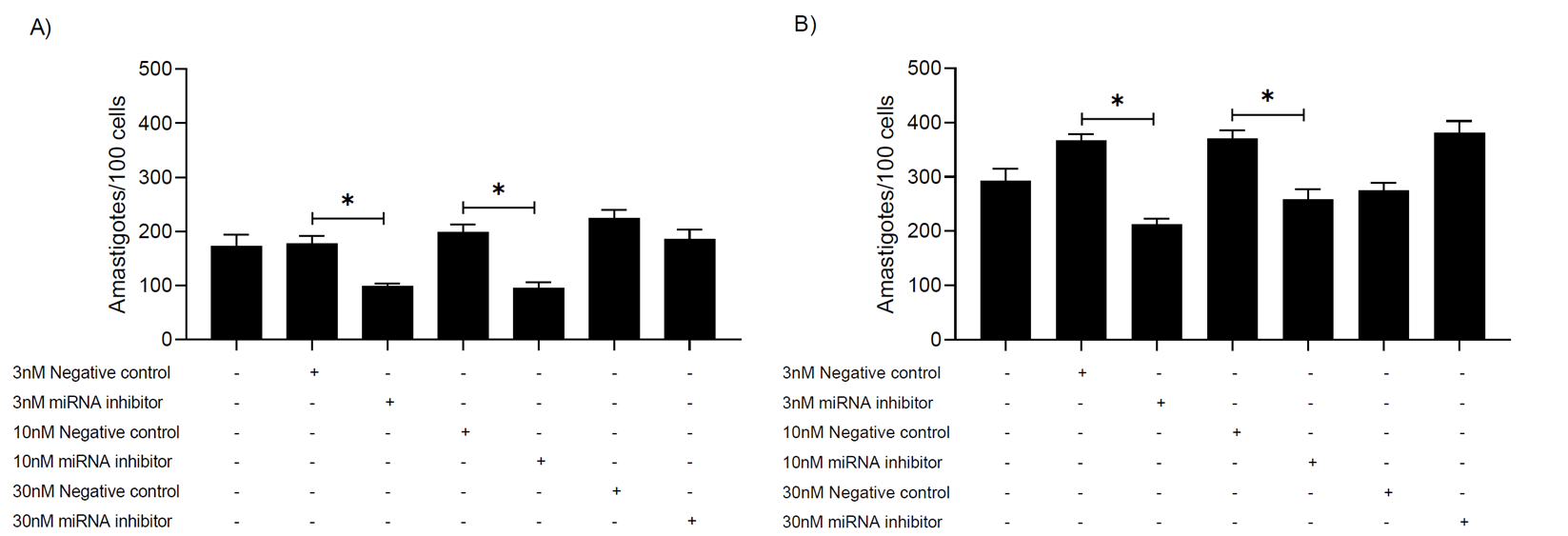

Supplement: Supplementary Figure 1 — Effect of different concentrations of miR-548d-3p inhibitor or negative control (scrambled miRNA) transiently transfected on parasite load (number of amastigotes/100 cells) in L. (V.) braziliensis promastigote-infected THP-1 cells at 6 h (A) and 24 h (B) post-infection. The experiment was carried out by adding the synthetic molecules at 3nM, 10nM or 30nM with the transfection reagent diluted in RPMI medium or only RPMI medium (non-transfected cells) to wells containing 106 THP-1 adherent cells and maintained for 24 h at 37°C (5% CO2) then infected with L. (V.) braziliensis promastigotes. (A) * = P < 0.05 (one way ANOVA and student t test). [file Image_1.tif]
